# Mapping Alzheimer’s disease heterogeneity through exploratory unsupervised learning

**DOI:** 10.3389/fnagi.2026.1869804

**Published:** 2026-07-16

**Authors:** Catarina Xavier, Ana Paula Correia, Joana Lopes, Joana Fernandes, Inês Laranjinha, Nádia Pinto

**Affiliations:** 1 i3S - Instituto de Investigação e Inovação em Saúde, Universidade do Porto, Porto, Portugal; 2 ULSSA - Unidade Local de Saúde de Santo António, Porto, Portugal; 3 IPATIMUP - Instituto de Patologia e Imunologia Molecular da Universidade do Porto, Porto, Portugal; 4 Centro de Matemática da Universidade do Porto (CMUP), Porto, Portugal

**Keywords:** Alzheimer’s disease, clustering algorithms, machine learning, subtyping, unsupervised learning

## Abstract

**Introduction:**

Alzheimer’s disease (AD) is becoming one of the most pressing health challenges of the century, affecting circa 55 million people worldwide and expected to triple this number by 2050. Besides the growing prevalence, AD remains difficult to diagnose due to its long preclinical phase and substantial symptomatic heterogeneity. Despite the existence of international guidelines for AD diagnosis emphasizing the use of biomarkers (such as neuroimaging and cerebrospinal molecular biomarkers), most research studies are still using data disregarding their diagnostic quality, blurring the overall statistical outcomes. Aiming to circumvent this effect, biomarker confirmed samples from the Alzheimer’s Disease Neuroimaging Initiative (ADNI) were used, which integrate genetic and neuroimaging data for all participants.

**Methods:**

Unsupervised machine learning clustering methods were tested to explore whether curated genetic markers can reveal underlying substructure within sporadic AD (sAD). Here two specific sets of single nucleotide polymorphisms (SNPs) were selected: (i) candidate SNPs highlighted in previous studies that identified the existence of different subtypes in sAD and (ii) SNPs found associated with sAD in genome wide association studies (GWAS) that used at least 50% of samples with biomarker-confirmed diagnosis.

**Results:**

This strategy minimized background noise and enabled the construction of high quality SNP sets for analysis. Different clustering algorithms were evaluated with agglomerative hierarchical clustering consistently yielding the most robust performance. Across SNP sets, most trials revealed a reproducible binary structure within sAD samples, suggesting the presence of genetically distinguishable subgroups.

**Discussion:**

These findings align with emerging multimodal evidence supporting biological heterogeneity in AD. Overall, this work demonstrates that curated SNP panels combined with unsupervised learning can uncover meaningful substructure in sAD, reinforcing the value of integrating high quality genetic data into subtype research.

## Introduction

Alzheimer’s disease (AD) is the major cause of dementia, affecting approximately 55 million people worldwide. As a slow progressive neurodegenerative disorder leading to loss of memory, loss of executive function and even behavior alterations, it is a devastating disease for patients, families and caretakers. AD’s diagnosis in an early phase may be challenging due to the long asymptomatic period and the variability of symptomatic subtypes. Current guidelines for the clinical diagnosis of AD recommend the use of biomarkers such as imaging and cerebrospinal protein signals to biologically confirm or rule out the disease (Jack et al., 2024), in line with those previously developed for research purposes (McKhann et al., 2011). Nonetheless, the sampling criteria of subjects used in AD were not globally implemented, as previously discussed (Escott-Price and Hardy, 2022; Xavier and Pinto, 2025). AD is a heterogeneous condition both in terms of clinical presentation and progression, suggesting distinct pathophysiological pathways that eventually led to the emergence of several subtypes, concerning different criteria (Table 1) that are sometimes interchangeable.

**TABLE 1:** List of AD subtypes according to different criteria, presenting a short description and the genetic variants associated, when possible.

Criteria	Subtype	Short description	Associated genetic variant(s)	References
Clinical observation	Typical (amnestic)	Predominant memory impairment; most common AD phenotype	*APOE*	Corder et al., 1993
	Posterior cortical atrophy	Visual and spatial processing deficits; occipital involvement	*APOE*	Schott et al., 2016
	Logopenic progressive aphasia	Language deficits with word-finding difficulty	*GRN* and *C9orf72*	Ramos et al., 2019
	Behavioral / dysexecutive	Behavioral and executive dysfunction	n.a.	–
Tau progression, brain atrophy	Typical AD	Tau pathology and atrophy located in hippocampus and association cortex. Most frequent subtype.	*MAPT, APOE*	Ferreira et al., 2020
	Limbic predominant	Tau starts in hippocampus and entorhinal cortex	*MAPT, TDP-43, APOE*	Na et al., 2016; Ferreira et al., 2020
	Hippocampal sparing	Neocortical tau burden with spared hippocampus; rapid progression	non-*APOE* ε4	Ferreira et al., 2020
	Minimal tau	Low total tau pathology despite cognitive decline	n.a.	–
Proteomics	Hyperplasticity	Elevated synaptic plasticity-related proteins	*TREM2, LILRB2, RHOH, APP*	Tijms et al., 2024
	Innate immune activation	Increased microglial/inflammatory protein markers	*IDUA, CLNK, SCIMP*	Tijms et al., 2024
	RNA dysregulation	Abnormal levels of RNA- binding proteins and ribonucleoproteins	*KAT8, BIN1, SNX1, TREM2, SPDYE3*	Tijms et al., 2024
	Choroid plexus dysfunction	Elevated levels of proteins with high expression in the lateral ventricle choroid plexus	*CLNK, ABCA7, PICALM, IL34*	Tijms et al., 2024
	Blood-brain barrier (BBB) dysfunction	Presence of blood proteins in the brain caused by a BBB leakage	APOE*, IL34, ECHDC3, APP	Tijms et al., 2024
Genetics	Familial AD	Caused by mutations in amyloid processing genes; early onset; mendelian,	*APP, PSEN1, PSEN2*	Tanzi, 2012
	*APOE* ε4 Risk	Late-onset AD subtype with increased amyloid and tau burden	*APOE*	Bertram et al., 2008
	Polygenic risk	Broad common variant risk across immune, synaptic, and lipid pathways	Several	–

*Higher frequency of cases but not statistically significant.

Regarding genetics, AD is classified into two major types: (i). familial AD, caused by very rare but with high-penetrance Mendelian mutations in specific genes: *APP*, *PSEN1*, and *PSEN2*, that typically cause early-onset disease, and (i)i. sporadic AD (herein sAD), the most common form [>99% of AD cases and 60%–80% of dementia ones (Alzheimers Dementia, 2023, 2025)], or “non-familial” in the strict monogenic sense, driven by many common variants of small effect. Despite the extensive research effort in sAD genetics, little is still unveiled. Although dozens of variants are identified by the more recent genome wide association studies (GWAS) (Wightman et al., 2021; Bellenguez et al., 2022), among these, the most impactful on sAD development is by large the ε4 variant of apolipoprotein E gene (*APOE*). Showing a variable distribution across populations, *APOE* ε4 isoform is considered the ancestral allele from which ε3 and ε2 evolved, yet today ε3 is the most frequent globally and ε4 is usually the second-most common (Singh et al., 2006). Interestingly, a north-south gradient was identified for ε4 in Europe with higher frequencies in northern regions decreasing toward southernmost countries (Singh et al., 2006; Brainstorm Consortium et al., 2018; Belloy et al., 2019) and showing different frequency patterns in Chinese and African populations (Singh et al., 2006; Hu et al., 2011; Hao et al., 2022).

Despite the massive increase in sample numbers, particularly with the most recent GWAS surpassing one million samples, the effect sizes of identified loci have remained modest. Although more than 70 loci are now identified as linked to developing sAD (Wightman et al., 2021; Bellenguez et al., 2022), their statistical impact with higher number of samples was not increased (Escott-Price and Hardy, 2022; Xavier and Pinto, 2025). A major contributor is the inconsistent application of rigorous diagnostic criteria across studies, which compromises case–control definitions and blurs genetic associations. sAD’s challenging diagnosis and long preclinical stages lead to poor construction of genomic databases that include individuals - both cases and controls, based on (i). single clinical appreciations [without biomarkers as suggested by recent guidelines (McKhann et al., 2011; Jack et al., 2024)], (ii). self- or third-party declarations of sAD status, and even (iii). familiar presence/absence of sAD. In fact, as shown by our previous review (Xavier and Pinto, 2025), only four GWAS presented >50% of samples selected with sAD confirmation/absence based on biomarkers following the accepted international guidelines. The Alzheimer’s Disease Neuroimaging Initiative database (ADNI) joins genetic and neuroimaging (MRI) data from all subjects, guaranteeing a confirmed diagnosis (or absence of the disease) for all samples present at the database. This is a clear cut from most databases that rely on non-confirmatory diagnosis such as clinical interviews (Xavier and Pinto, 2025). However, other AD subtypes beyond familial/sporadic based on genetic markers analyses have been described and characterized extensively within other fields of research and medicine.

Clinical AD phenotypic variants are usually defined by their clinical symptoms (Graff-Radford et al., 2021) or cognitive performance (Morris et al., 2014). The amnesic syndrome is by far the most common and well-known type of AD. Other subtypes include non-amnesic-predominant variations, such as posterior cortical atrophy known for presenting complex visual impairments as one of the initial symptoms (Crutch et al., 2017; Sahoo et al., 2018), logopenic variant primary progressive aphasia presenting language difficulties (Ahmed et al., 2012; Crutch et al., 2017; Spinelli et al., 2017), and behavioral/dysexecutive that is characterized by executive function impairment and behavioral alterations (Rascovsky et al., 2011; Ossenkoppele et al., 2015). Although *APOE* ε4 allele has been linked to an increased risk for typical AD and posterior cortical atrophy, logopenic variant primary progressive aphasia showed a different genetic constellation, including different variants in *GRN* and *C9orf72* genes (Ramos et al., 2019; Samra et al., 2023).

Research into neuroimaging data (PET, MRI) normally describes AD in terms of tau progression or accumulation patterns and brain atrophy locations, in what is commonly called AD biological subtypes (Ferreira et al., 2020). Four major AD subtypes are recurrently identified: typical, limbic-predominant, hippocampal-sparing and minimal atrophy. Ferreira et al. (2020) describes two major dimensions of heterogeneity: severity and typicality. Typical AD and minimal atrophy are two subtypes in the extremities of the severity axis, whereas limbic-predominant, typical AD and hippocampal-sparing vary within the typicality axis. Typical AD presents tau-accumulation and brain atrophy in hippocampus and association cortex and is the most frequent of the subtypes, accounting for circa 55% of cases. Despite showing residual brain atrophy, the minimal atrophy subtype displayed the highest cerebrospinal (CSF)-tau altered values, suggesting molecular-level neurodegeneration as a driver of symptoms (Rascovsky et al., 2011; Ferreira et al., 2020). Limbic-predominant presented more affected females and *APOE* ε4 carriers and showed later onsets, whereas hippocampal-sparing was more frequent in males and non *APOE* ε4 carriers and was correlated with earlier onsets.

Proteomics research of AD focuses mostly on the analysis of CSF proteins recurring to mass spectrometry techniques and antibody-based immune assays. Recent efforts in this field of study have recently identified five molecular AD subtypes (Tijms et al., 2024). Neuronal hyperplasticity subtype is linked to the presence of synaptic plasticity proteins and correlated with earlier onset sAD with fast progression. Innate immune activation subtype is associated with an increased representativity of inflammation-related proteins, more frequent in patients with older onsets of the disease and APOE ε4 carriers with substantial brain atrophy. RNA dysregulation subtype is associated with abnormal levels of RNA binding proteins and correlated with an increased cognitive impairment. Choroid plexus dysfunction indicates barrier breakdown and CSF dysregulation (including transporter and secretory proteins from plexus cells) and finally blood-brain barrier dysfunction shows a leakage of proteins from plasma into CSF, a subtype that is more frequent in older patients, which shows an increased severity with vascular comorbidities. These subtypes also associate with specific genetic variants (Tijms et al., 2024) (Table 1), reinforcing the need for interdisciplinary approaches to fully characterize sAD.

The application of multimodal analyses to sAD research is emerging as a relevant interdisciplinary approach, integrating clinical observation, neuroimaging, genetics and other data into machine learning algorithms that predict the risk for sAD. For a thorough review of multimodal approaches for sAD please consult Wahul et al. (2025). Since 2023, this trend has increased in publications and development of successful tools for sAD subtyping using various combinations of multimodal datatypes (MRI, PET, and others), of which some used genetics (Varol et al., 2017; Tijms et al., 2024; Yang et al., 2024). A recent multimodal study on sAD prevention when adopting healthier lifestyles was also published recently (Barbera et al., 2023). This highlights alternative usages for multimodal predictive algorithms in sAD phenotyping.

Integrating these different subtyping perspectives requires a multiscale analytical strategy. Here, different unsupervised machine learning methods are applied to a set of curated samples to explore sAD subtyping using SNPs from biomarker-confirmed GWAS. Preliminary analyses using candidate markers and unsupervised clustering algorithms support the feasibility of genotypic AD subtyping.

## Material and methods

### Samples and SNPs selection

Data used in the preparation of this article were obtained from the Alzheimer’s Disease Neuroimaging Initiative (ADNI) database^1^. The ADNI was launched in 2003 as a public-private partnership, led by Principal Investigator Michael W. Weiner, MD. The primary goal of ADNI has been to test whether serial magnetic resonance imaging (MRI), positron emission tomography (PET), other biological markers, and clinical and neuropsychological assessment can be combined to measure the progression of mild cognitive impairment (MCI) and early Alzheimer’s disease (AD). Samples were retrieved from three ADNI GWAS initiatives: 1, GO, and 2 (see text footnote 1) and filtered for sAD diagnosis. All subjects were genotyped using different Illumina SNP arrays: Human610-Quad, HumanOmniExpress, and Omni 2.5M chips, as previously described by Saykin et al. (2010). Since only ADNI was used as source of genetic data, the sAD diagnosis was confirmed with neuroimaging biomarkers, avoiding the introduction of dubious data as seen in recent AI-led biomedical studies (Basu, 2026).

Single nucleotide polymorphisms selection followed three major criteria: (1) SNPs identified in publications describing sAD subtypes, including the proteomics study described in Tijms et al. (2024) and multimodal genetics-imaging studies; (2) SNPs associated with increased sAD risk in genetic association studies with >50% biomarker confirmed cases/controls (Seshadri et al., 2010; Jun et al., 2014; Sherva et al., 2014; Herold et al., 2016) and (3) the APOE defining SNPs (Weisgraber et al., 1981; Rall et al., 1983). An initial list of 69 SNPs was compiled (Supplementary Table 1). Of these, 45 SNPs were absent from ADNI’s genotyping arrays, including the APOE-defining allele variant rs429358. Proximal SNPs (<50 bp with highest minor allele frequency) were sought as substitutes for all missing SNPs, and only rs1053504 was found, serving as a proxy for rs2245466 (Tijms et al., 2024).

Finally, three SNP subsets were formed for analysis as follows: Group 1 consisting of 9 previously signalized subtype-SNPs, Group 2 based on 18 GWAS risk-SNPs, and Group 3 composed of combined 24 SNPs (Supplementary Table 2). Due to high missingness in rs10456232 (hereafter “Miss_SNP”) within Group 2, subsets were analyzed both with and without this marker. Additionally, owing to the known impact of rs7412 (*APOE*-defining allele, with rs429358 absent from data), separate analyses were performed including or excluding rs7412 (hereafter designated “*APOE*2”).

Genotypes were encoded as 0/1/2 using a consistent reference allele. When no ancestral allele was annotated in Ensembl^2^, the reference allele was arbitrarily but deterministically selected and then used consistently for all individuals and all clustering runs, following standard practice in population genetics- analyses (Patterson et al., 2006; Price et al., 2006; Purcell et al., 2007). Because the analyses rely on relative genotype coding (0, 1, 2) rather than on the biological interpretation of ancestral versus derived alleles, reversing the coding for a given SNP (0↔2) does not alter the underlying genotype relationships and is not expected to affect clustering stability.

Despite several SNPs appearing to be tri- or even tetra-allelic (Supplementary Tables 1, 2), no evidence was found in the current set of samples and therefore, the same numeric system (0, 1, and 2) was applied to all markers. All samples that presented at least one missing value were excluded from the analysis, and all duplicates were excluded. SNPs with missing values above 10% were excluded from analysis, following widely used quality control practices in -human genetics- studies. A 10% SNP level- missingness threshold is recommended in standard GWAS QC pipelines (Anderson et al., 2010; Saykin et al., 2010) and has been applied in large consortia datasets including HapMap and early 1000 Genomes releases (Abecasis et al., 2010; Altshuler et al., 2010). This threshold improves robustness by removing poorly genotyped markers, but a more permissive cutoff would retain additional SNPs at the cost of increased genotype error and potentially less stable downstream clustering, particularly if missingness is non-random. It is therefore considered the 10% threshold a reasonable compromise between marker retention and data quality. A final set was selected for further analysis, only with sAD confirmed samples according to ADNI latest medical reports; final numbers varied per selected SNP set and can be consulted in Table 2.

**TABLE 2:** Best scoring algorithms/conditions per group considering the Silhouette score (variable within −1 and 1 interval).

SNP set	*N*	# SNPs	Algorithm	k	Silhouette [−1;1]	CH	DB	SNPs (RFI value)
Group 1	1,167	9	Agglomerative	2	0.309	178,939	1,373	rs724585 (0.946)
Group 1 + *APOE*2	788	10	Agglomerative	3	0.281	97,445	1,431	rs724585 (0.495), rs7412 (0.404)
Group 2	669	18	Gaussian mixture	2	0.245*	54,410	2,510	rs2421847 (0.305), rs1861525 (0.487)
Group 2–Miss_SNP	1,110	17	Agglomerative	4	0.222	95,015	2,063	rs7245858 (0.223), rs2421847 (0.190), rs1861525 (0.248), rs11154851 (0.185)
Group 2 + *APOE*2	380	19	Agglomerative	4	0.258	24,498	1,521	rs7245858 (0.318), rs7412 (0.185), rs2421847 (0.149)
Group 2–Miss_SNP + *APOE*2	779	18	KMeans	2	0.244*	53,697	1,612	rs2421847 (0.743)
All SNPs–Miss_SNP	1,318	24	Spectral	2	0.198	17,976	1,237	rs7245858 (0.172), rs11154851 (0.257)
All SNPs–Miss_SNP + *APOE*2	773	25	KMeans	2	0.210*	38,545	2,138	rs2421847 (0.612)

*Reflect the highest scores obtained for a single iteration. Calinski-Harabasz (CH) and Davies-Bouldin (DB) scores were also tested per trial. Single nucleotide polymorphisms (SNPs) with random forest feature importance (RFI) results > 0.1 are listed in the last column, RFI values within brackets.

### Clustering and statistical analyses

Clustering analyses were performed in Python 3 using Spyder IDE v. 5.1.5 and scikit-learn library, following standard guidelines (code provided as Supplementary File 1). For this study, different clustering algorithms were tested: one deterministic (same outcome independent of the number of runs - DBSCAN), and four stochastic [different outcomes with different runs - K-means, agglomerative hierarchical clustering, spectral clustering (nearest neighbor – NN), and gaussian mixture model (GMM)]. For stochastic methods, the number of clusters (k) ranged from 2 to 5, with three independent iterations for each case. For DBSCAN, various epsilon (eps, distance threshold) and minimum samples (core points threshold) were systematically tested (Supplementary Table 3).

Clustering quality was evaluated using three complementary internal validation metrics: (i). silhouette score (range −1 to 1; higher values indicate better-defined clusters with strong intra-cluster cohesion and inter-cluster separation); (ii). Calinski-Harabasz index (CH, positive, unbounded; higher values indicate more compact and well-separated clusters, though sensitive to outliers); and (iii). Davies-Bouldin index (DB, positive, unbounded; lower values indicate better cluster separation and distinctness). Comprehensive validation metrics for all algorithms, runs, sample sets, and k values are provided in Supplementary Table 3. To gauge the SNP-specific relevance in determining the creation of the different clusters, two metrics were calculated: (i). random forest feature importance (RFI) derived from a classifier trained to predict cluster labels, and ii. mutual information (MI) between individual SNPs and cluster assignments. SNPs with RFI ≥ 0.1 are highlighted as high-impact drivers in Table 2; full values for all SNPs across all groups are in Supplementary Table 4.

**FIGURE 1:**
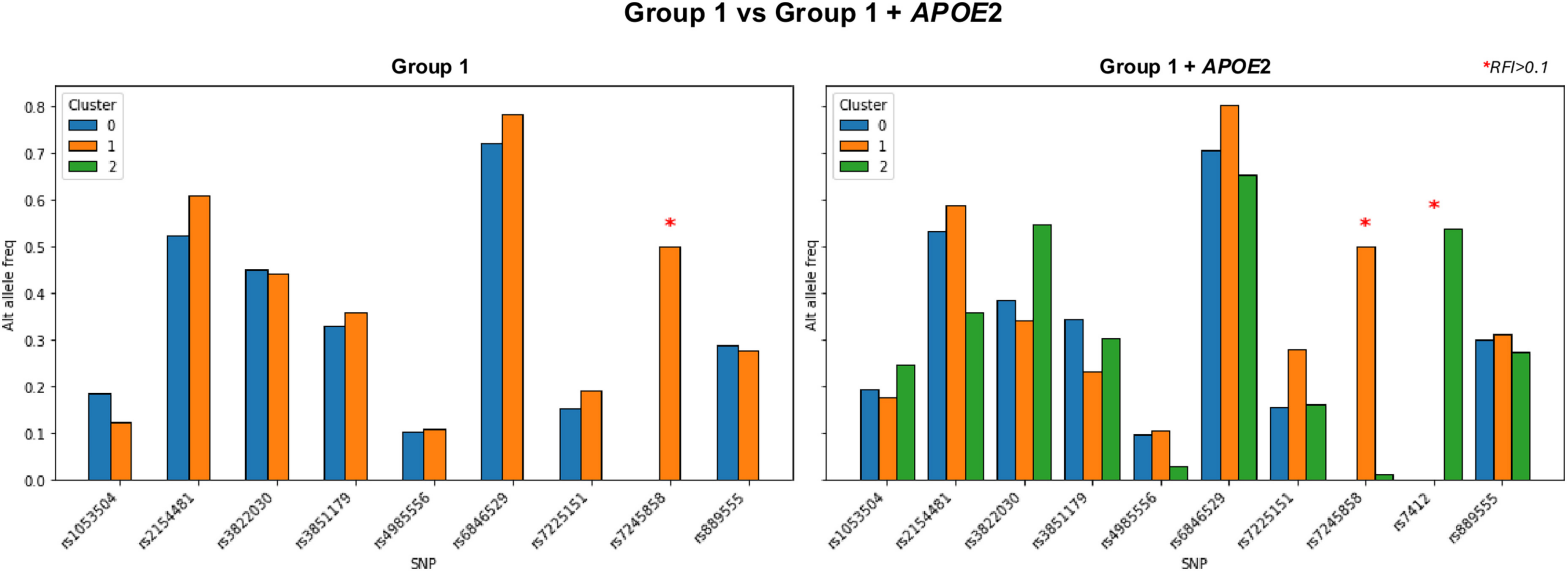
Alternative allele frequencies per cluster (using the best scoring scenario) formed for Group 1 and Group 1 + *APOE*2 sets. SNPs with RFI > 0.1 are highlighted with a red asterisk.

**FIGURE 2:**
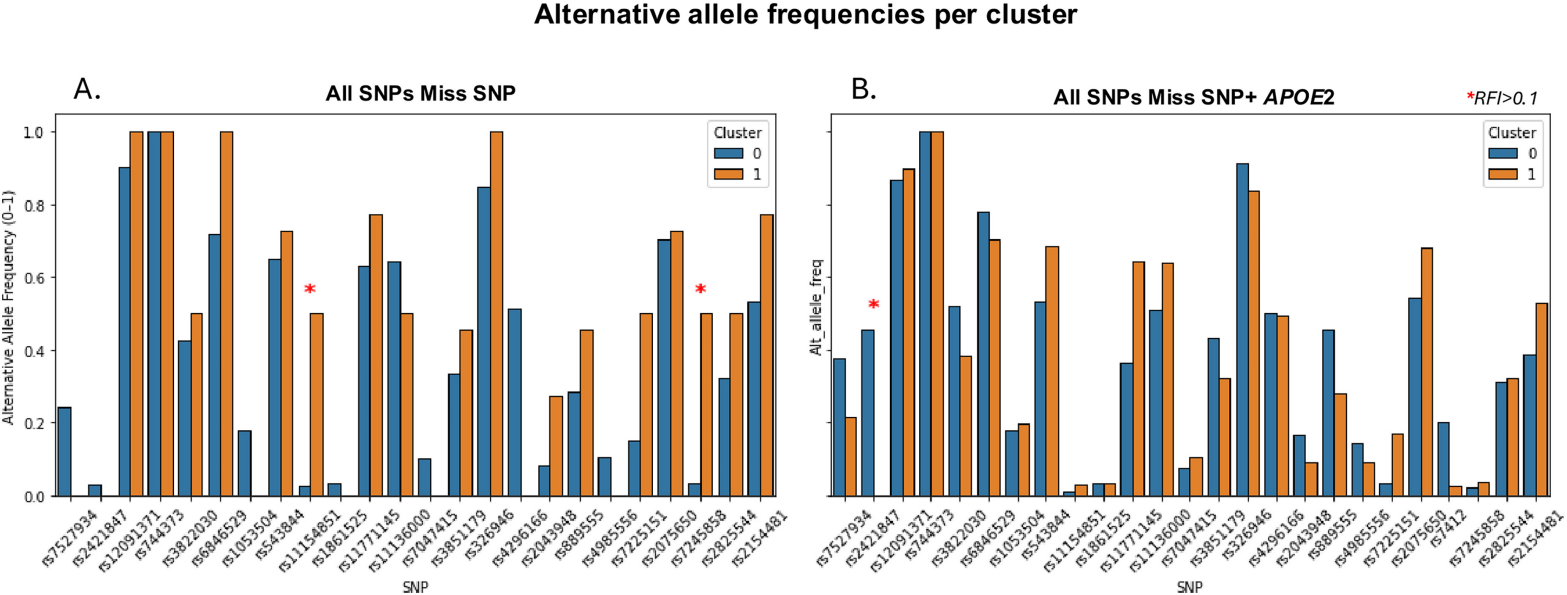
Alternative allele frequencies per cluster (using the best scoring scenario) formed for all sets within Group 2: Group 2, Group 2 + *APOE*2, Group 2 Miss SNP, and Group 2 Miss SNP + *APOE*2. SNPs with RFI > 0.1 are highlighted with a red asterisk.

All visualizations were created with plotly, matplotlib, and seaborn. Allele frequency distributions across clusters were visualized as grouped barplots for *APOE*2 containing/non-containing SNP sets (Figures 1, 2). Sankey diagrams (Supplementary Figure 1) depict sample flows between clusters in *APOE*2 containing/non-containing analyses, revealing clustering stability. Co-clustering heatmaps (Supplementary Figure 2) display pairwise concordance of sample groupings across different SNP set trials.

## Results

### Analysis of SNP subsets

Each one of the SNP subsets was evaluated using multiple clustering algorithms and settings in a systematic manner. Stochastic methods were run for k = 2–5 clusters, across three iterations; while the deterministic DBSCAN algorithm was optimized by testing various epsilon (distance threshold) and minimum samples (core points) parameters. The obtained results consistently support the existence of clusters within sAD samples, across all SNP subsets (despite quality of clustering varies by set). Full internal clustering validation metrics across runs, settings and algorithms that produced at least two clusters can be consulted in the Supplementary Table 3. The best scoring combination (algorithm/settings) per SNP subset is displayed in Table 2. Best per SNP subset was selected considering the highest Silhouette score.

Group 1 analysis of 9 subtyping-associated SNPs (Supplementary Table 2) revealed a robust two-cluster structure in biomarker-confirmed sAD cases (Table 2 and Supplementary Table 3), with optimal performance from agglomerative clustering at k = 2 (silhouette = 0.309, CH = 178.9, DB = 1.37) and concordant results from K-means, GMM, and DBSCAN. Alternative-allele frequency plots for Group 1 (Figure 1A) show a marked bimodality at rs7245858, with one cluster exhibiting high alternative-allele frequency and the other low-to-absent, consistent with its overwhelming random-forest importance (RFI = 0.9455, MI = 0.2516; Supplementary Table 4), whereas the remaining SNPs display only modest contributions. When APOE2 (rs7412) is added (Group 1 APOE2; Supplementary Table 3), the same two-cluster topology is preserved (silhouette = 0.261), with rs7245858 and APOE2 emerging as the main drivers (RFI = 0.4949 and 0.4042, respectively; Supplementary Table 4), indicating that APOE refines but does not redefine the underlying structure. Crucially, pairwise LD estimated using LDlink v5.3LD (Machiela and Chanock, 2015), for European population (matching major ADNI biogeographic ancestry) demonstrates that these SNPs, both located at chromosome 19, are effectively independent (r^2^ = 0.002, D’ = 0.064), reinforcing rs7245858 as a non-APOE signal. Taken together - and considering that several Group 1 loci were originally selected from proteomic subtype studies related to innate immune activation, choroid plexus dysfunction, and RNA dysregulation - these results support the existence of two reproducible, biologically interpretable genotypic sAD subtypes, whose stability across SNP sets is further illustrated by the Group 1 to Group 1 + APOE2 cluster flows in the Supplementary Figure 1.

Group 2 reveals moderate but consistent polygenic substructure in sAD, contrasting Group 1’s pair-SNP dominance. Due to high missingness values in rs10456232 (Miss_SNP), Group 2 clustering was tested with and without this SNP, nearly doubling sample size upon removal (*N* = 669→1,110). Peak silhouette scores varied across subsets (Table 2), but agglomerative clustering consistently ranked among the top performers and showed high stability across iterations, matching patterns observed for Group 1 + *APOE*2 (Supplementary Table 3) Gaussian Mixture Models peaked for full Group 2 (k = 2; silhouette = 0.245), though iteration variability was notable. For the Miss_SNP-excluded set, agglomerative clustering (k = 4; silhouette = 0.222) outperformed K-means (k = 2; 0.220). These results point toward the agglomerative algorithm as being the more robust clustering method across conditions for these datasets. Noteworthy, excluding Miss_SNP (rs10456232) nearly doubled sample size, markedly improved cluster compactness (CH: 61.5→95.0, +54.6%), and enhanced solution reproducibility (Table 2 and Supplementary Table 3), despite its negligible contribution to clustering (RFI = 0.000; Supplementary Table 4). Supplementary Figure 1C reveal increased cluster flux upon *APOE*2 addition to Group 2 vs. Group 1 stability (Supplementary Figure 1): B (with Miss_SNP: largest cluster without *APOE*2 shifts primarily to one cluster with *APOE*2), C (without Miss_SNP: two major clusters without *APOE*2 redistribute to one dominant cluster with *APOE*2), with 65%–75% of samples retaining their genetic identity across partitions - demonstrating that risk-based subtypes endure and are refined (not destroyed) by *APOE*2. Ultimately, polygenic clusters proved durable, refined rather than erased by additional genotyping data - a hallmark of robust subtyping. Five SNPs showed RFI > 0.1 (Table 2) for at least one subset: rs2421847, rs1861525, rs7245858, rs7412 (*APOE*2), and rs11154851. Only rs2421847 demonstrated consistent importance across all Group 2 conditions while rs10456232 (Miss_SNP) exclusion was required to unmask the full polygenic structure (Supplementary Table 4).

Indeed, rs2421847 was the most consistent Group 2 driver, exceeding RFI > 0.1 across all four subsets (Supplementary Table 4), with statistically differential allele frequencies between clusters (Figure 2). The alternative allele of rs2421847 was present in one of the clusters for all sets in Group 2 constellations, varying between 0.25 and 0.5 in frequency and neglectable frequencies in the remaining clusters (<0.024). Its importance peaked at RFI = 0.743 in the MissSNP-excluded + *APOE*2 analysis, ranging 0.149–0.743 regardless of data quality (+/− Miss_SNP) or *APOE*2 inclusion, outperforming all other markers (Supplementary Table 4).

Rs1861525 serves as a cautionary example of a noise-sensitive SNP that appears dominant in raw data but reveals its fragility through systematic confounder analysis. In the full Group 2 dataset (with Miss_SNP), it led clustering with RFI = 0.487 (#1) but excluding Miss_SNP dropped 49% to RFI = 0.247 (still #1), indicating partial reliance on imputation artifacts from low-quality genotypes. Adding *APOE*2 further collapsed its RFI to ≤0.016 due to *APOE*’s homogenizing effect in tight LD with ancestry markers, which compresses rs1861525’s gradient and masks its subtler signal (Figure 2). Unlike stable drivers like rs2421847 and rs11154851 this context-dependence - coupled with stable MI = 0.172–0.225 - proves rs1861525 is not a “real” universal cluster driver but an artifact amplified by MissSNP noise and overridden by *APOE*2 stratification. Such sensitivity underscores the necessity of multi-step validation (MissSNP exclusion, *APOE* stratification) to distinguish genuine ancestry markers from methodological illusions, positioning this thorough approach as essential for robust SNP selection in Alzheimer’s genomic studies (Supplementary Table 4). Indeed, group-stratified analyses revealed distinct SNP hierarchies also for rs7245858 that dominated Group 1 clustering but showed limited influence in Group 2 (RFI = 0.0060 in raw data; 0.2226 when – Miss_SNP, and 0.0033 when +*APOE*2), exemplifying panel-optimized, population-specific ancestry signals (Supplementary Table 4). Conversely, *APOE*2 (rs7412) emerged as a potent confounder across panels, rising to RFI = 0.4042 (#1 in G1, surpassing rs7245858) and 0.1853 (top-3 in G2), homogenizing clusters while suppressing other markers. This hierarchical resolution: Miss_SNP exclusion then *APOE* stratification distinguishes true ancestry drivers from methodological artifacts, validating the multi-step approach essential for robust sAD genomic subtyping (Figure 2 and Supplementary Table 4).

### Analysis of the combined SNP datasets

Aiming to understand whether better results were obtained when both sets of SNPs were combined for analysis, both Group 1 and Group 2 test SNPs (with and without the *APOE* SNP and excluding Miss_SNP: rs10456232, due to its demonstrated noise amplification) were joined for clustering tests. Full clustering trial results can be observed in Supplementary Table 3 and the best scoring results are depicted in Table 2.

A closer inspection of runs and both subsets allows observing that silhouette scores for k = 2 are on average 0.135 (for stochastic clustering algorithms) with a median of 0.153. Average and median values considering all clustering tests are markedly lower for other k values, showing consistency for all methods (Supplementary Table 3). Silhouette scores for DBSCAN trials that produced more than one cluster were very low (0.016) or in the case of the SNP set with rs7412 (*APOE*2), only one cluster was produced and thus, the method was discarded. The best clustering scenarios for both SNP sets were based on stochastic methods for k = 2 with similar silhouette scores (0.198 for all SNPs and 0.210 when including *APOE*2). Once again, despite observing in the group +*APOE*2 that the highest scoring algorithm was the KMeans (k = 2), it did not present as consistent results in the replicates as the agglomerative algorithm for k = 2 (the second highest score). Indeed, these results reinforce that the agglomerative clustering method is the most appropriate for this dataset. Consistently with the clustering metrics, the co-occurrence heatmap for the all-SNP panel (Supplementary Figure 2) shows that most individual pairs co-cluster in both analyses (with and without *APOE*2), with co-occurrence frequencies close to 1, indicating that *APOE*2 inclusion only induces limited local reassignment rather than a global reorganization of cluster structure.

Group 3 combined SNP analyses confirm that SNP dilution weakens clustering across all algorithms compared to optimized Group 1/2 panels. In Group 3 (−Miss_SNP), Spectral clustering achieved the highest silhouette of 0.198 (k = 2, CH = 66.1, DB = 3.107), outperforming KMeans (0.168), Agglomerative (0.152), and Gaussian Mixture (0.155), but all trailed Group 1’s Agglomerative solution (0.309, CH = 179, DB = 1.373) and Group 2’s KMeans leader (0.234, CH = 61.0). *APOE*2 stratification shifted Group 3 dominance to KMeans (0.210, k = 2), with Agglomerative close (0.180), while Spectral collapsed to −0.021—demonstrating algorithm-specific confounder sensitivity—and CH scores decreased from 66.1 to 38.5, confirming that homogenization reduced separation power. Algorithm rankings reveal Agglomerative’s Group 1 specialization, KMeans’ G2/G3 + *APOE* versatility, Spectral’s raw multi-panel capability (but *APOE* fragility), and GMM’s consistent weakness. These results validate Miss_SNP exclusion (improved metrics throughout) while showing that smaller, panel-optimized datasets with appropriate algorithm choice yield superior sAD subtyping compared with redundant 24-SNP combinations (Supplementary Table 3). Supplementary Figure 1 Sankey plots illustrate that *APOE*2 inclusion leaves Group 1 clusters largely stable, destabilizes Group 2 when rs10456232 is present but not after its removal, and induces mostly symmetric but low-information cluster reshuffling in the all-SNP setting, consistent with SNP dilution and the benefits of Miss_SNP cleaning (Supplementary Figure 1).

**FIGURE 3:**
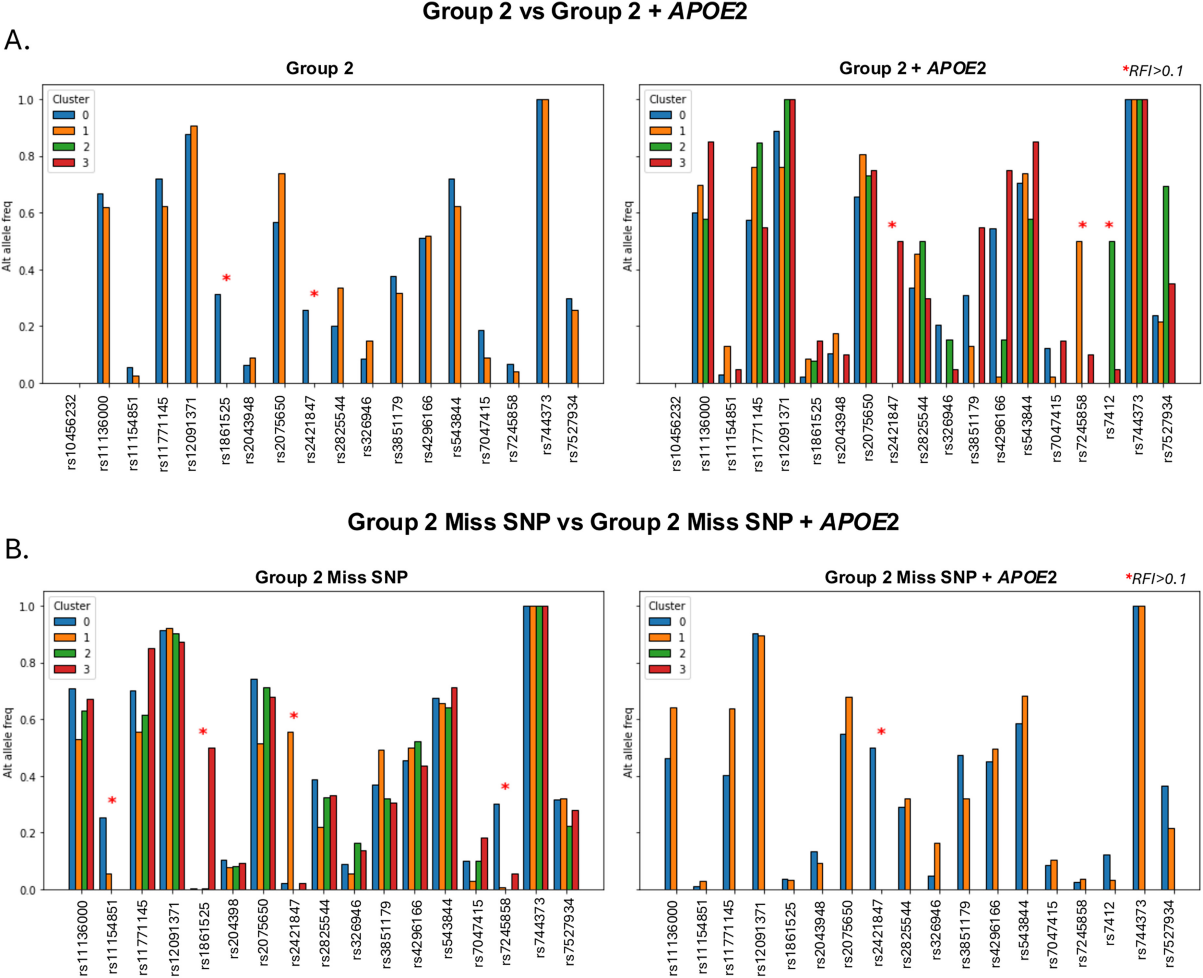
Alternative allele frequencies per cluster (using the best scoring scenario) formed for both sets of “All SNPs”: **(A)** All SNPs Miss SNP and **(B)** All SNPs Miss SNP + *APOE*2. SNPs with RFI > 0.1 are highlighted with a red asterisk.

For the All SNPs dataset (Group 1 + Group 2 SNPs, excluding Miss_SNP rs10456232), two SNPs presented RFI values above 0.1: rs11154851 (*PDE7B/LOC644135*) and rs7245858 (*LOC390956/ZDHHC11B*), both previously identified in Group 2 and, in case of the latter also in Group 1. These results highlight the importance of these two SNPs for cluster determination in this combined panel. When adding the *APOE* SNP, only one SNP shows RFI > 0.1 - rs2421847 (PRRC2C), mimicking the pattern observed for Group 2 – Miss_SNP + *APOE*2. Figure 3 depicts the alternative allele frequencies in both sets for all clusters and SNPs. In the non-*APOE*2 dataset (k = 2), rs11154851 shows an alternative allele frequency close to 0.5 in one cluster and about 0.027 in the other, whereas in the *APOE*2 subset the frequencies are ∼ 0.367 and 0.643. Rs7245858 also reaches ∼0.5 vs. ∼0.03 for the alternative allele in the two clusters of the non-*APOE*2 subset, but its frequencies drop to ∼0.02 and 0.04, in the *APOE*2 set, a pattern consistent with reduced sample size (from 1318 to 773, Table 2) and *APOE*2-driven homogenization. Although not reaching RFI > 0.1 in this context (RFI = 0.0564), the *APOE* SNP rs7412 still shows differential alternative allele frequencies between clusters in the All SNPs + *APOE*2 subset (∼0.20 vs. ∼0.03), supporting its role as a relevant, but mainly confounding, stratification factor rather than a primary cluster driver.

## Discussion

Biomedical genomic databases generally aggregate diverse patients under a broad phenotypic “”umbrella,” and AD is not only an exception, as even the proper definition of case/control may suffer greatly more than other diseases due to its nature and difficulty of the diagnosis. As previously discussed (Escott-Price and Hardy, 2022; Xavier and Pinto, 2025), the lack of standardized and rigorous criteria for including subjects in genomic databases that later are included in (non-independent) GWAs may be hampering the discovery of stronger genetic associations concerning sporadic AD. This affects not only cases, as also controls due to the long preclinical phase that can lead to (yet) non-symptomatic patients being used as controls (with corresponding wrongly considered “protective” genetics). Additionally, and this roots the development of this work, if AD heterogeneity reflects distinct molecular mechanisms with opposing genetic variants associated, pooling subtypes dilutes signals and weakens associations. Notwithstanding, besides the unquestioned evidence of heterogeneity, sparse or inconsistently structured metadata do not support the assessment of genomic subtyping, as corresponding phenotypic/clinical data are often unavailable or non-harmonized across resources. Given this, in this genetics-first exploratory approach using only the most reliable biomarker-confirmed candidate markers and genotype data, genetic-only subgroups were investigated as a foundation for subsequent multimodal integration in AD.

Alzheimer’s Disease Neuroimaging Initiative’s predominantly European ancestry cohort provides a homogeneity advantage for signal detection. Nevertheless, and despite the analysis being based on disease-selected SNPs rather than on ancestry-informative markers, the observed structure cannot be cleanly decomposed into disease-related and ancestry-related components. Accordingly, residual ancestry stratification cannot be excluded, although the predominantly European composition of the cohort likely reduces this risk. Indeed, this uniformity reduces ancestry confounding, aiding robust clustering. While multi-ancestry validation remains essential given the global AD burden, complete-case filtering implicitly controls subtle stratification. Future work should prioritize ancestry-stratified diverse cohorts (e.g., African/Asian ancestries) where *APOE* effects are known to differ and the same can occur for others. Although ADNI’s predominantly European ancestry composition provides a relatively homogeneous background that reduces stratification noise, it also limits the generalizability of our findings. No formal ancestry correction was applied because clustering framework relies on SNP level distances rather than regression modeling; however, subtle population structure may still influence cluster boundaries. Moreover, the main SNP drivers identified here were detected within a European LD and allele frequency context and may not behave similarly in non-European populations. Independent validation in diverse, biomarker confirmed cohorts is therefore essential. Future work should incorporate ancestry stratified clustering, LD aware evaluation of population specific proxies, and replication across multiple datasets to determine whether the observed genetic substructure reflects shared sAD biology or ancestry dependent patterns. Regarding the latter, it should however be noted that suitable AD databases are relatively scarce, in part because inclusion criteria vary substantially across studies and many datasets do not provide the biomarker-confirmed, harmonized multimodal data required for this analysis (Escott-Price and Hardy, 2022; Xavier and Pinto, 2025).

In this study, stratified clustering and feature-importance analyses identified a small subset of SNPs that consistently drive robust cluster structure in sporadic Alzheimer’s disease, while simultaneously revealing noise-sensitive and *APOE*-dependent artifacts. While rs429358 and rs7412 represent the principal *APOE* SNPs driving AD risk, the current panel includes only rs7412 (*APOE*2); nonetheless, this powerful variant was used to stratify samples into *APOE*2 + and *APOE*2 – subsets for analysis, isolating its confounding influence. Following standard GWAS and database quality-control guidelines, in this work a 10% missingness threshold was considered a reasonable compromise between retaining SNPs and minimizing the inclusion of poorly genotyped loci that could introduce noise into downstream analyses.

A three-tiered design probed how panel composition and preprocessing affect cluster structure: a focused Group 1 panel (previously identified subtyping-SNPs), a complementary Group 2 panel [GWAS risk SNPs, tested with and without rs10456232 (MissSNP) due to high missingness], and a pooled Group 3 “All SNPs” panel (Groups 1 + 2 excluding MissSNP, ±*APOE*2 stratification). The consistent preference for k = 2 across groups and algorithms further suggests that the dominant latent signal in this sAD cohort is essentially binary, with higher-order partitions mainly degrading cluster quality rather than uncovering stable subtypes. Algorithm behavior reinforces this view, as agglomerative clustering not only produced the best-separating solution in Group 1 but also showed the most stable performance in the noisier, *APOE*2-stratified all-SNP setting, whereas KMeans and Spectral were more sensitive to panel composition and *APOE*2, and DBSCAN proved ill-suited to the data manifold. The *APOE*2 experiments and the impact of rs10456232 highlight that biologically meaningful structure can coexist with strong confounding, so that *APOE* and noisy SNPs reshape partitions without substantially improving global separation, underscoring the need for careful panel curation and method selection rather than relying on raw dimensionality increases. This panel-level observation mirrors the pattern described previously at the GWAS level (Escott-Price and Hardy, 2022; Xavier and Pinto, 2025), where ever larger, but diagnostically heterogeneous, cohorts have been shown to dilute AD genetic signals and yield diminishing gains in effect sizes and explained heritability, emphasizing that in the genetics of complex traits “bigger” datasets - whether in subjects or SNPs - are not inherently “better” when added information is of lower quality or introduces additional heterogeneity. Clustering comprehensiveness is evidenced by Supplementary Table 3 reporting all metrics (silhouette, CH, DB), runs, and settings across five algorithms (k = 2–5, 3 stochastic replicates). Peak silhouette 0.309 exceeds typical genetic studies (0.2–0.3 range), confirmed by high CH = 178.9 and low DB = 1.37. Stability summaries show > 80% co-clustering (Supplementary Figure 2 heatmaps) and minimal flux between *APOE*2 ± runs (Sankey flows, Supplementary Figure 1). These multi-metric visuals validate reproducible binary structure beyond single measures. Although the obtained silhouette values represent a moderate separation, the stability of the -two cluster solution across algorithms, SNP subsets, and *-APOE* stratified- analyses suggests that the observed structure is reproducible rather than algorithm specific noise. Therefore these values can be interpreted as evidence of modest but consistent genetic substructure rather than strongly partitioned subtypes.

These findings on panel design and algorithm robustness naturally direct attention to the specific SNPs that proved most resilient to these challenges. Among the markers evaluated, rs2421847, rs7245858, rs11154851, and rs4296166 emerged as the most robust contributors to cluster structure across stratified analyses and preprocessing conditions, and therefore, merit prioritization for further functional and replication studies. This selection is based on convergent evidence from random-forest importance and clustering stability across multiple conditions. Rs2421847 consistently shows high RFI (≥0.1) in all four Group 2 subsets, peaking at 0.743 in the - Miss_SNP + *APOE*2 setting, where it becomes the dominant driver irrespective of data cleaning or *APOE*2 inclusion. Rs7245858 displays very high importance in Group 1 (RFI = 0.9455 in raw data and 0.4949 with *APOE*2) and remains non-negligible in Group 2 and in the combined All SNPs panel (RFI = 0.1724), indicating a reproducible effect beyond a single configuration. Rs11154851 behaves as a “cleaning-revealed” marker, rising from low importance in raw Group 2 to RFI = 0.1845 in Group 2 - Miss_SNP and 0.2570 (top ranked) in All SNPs - Miss_SNP, thus strengthening as noisy genotypes are removed rather than collapsing like rs1861525. Rs4296166 shows intermediate but stable importance (e.g., RFI≈0.07 in Group 2 + *APOE*2 and 0.0667 in All SNPs–Miss_SNP), never dropping to zero and retaining signal across preprocessing steps. In contrast, rs1861525 illustrates a fragile profile, with RFI falling from 0.487 (raw Group 2) to 0.247 after Miss_SNP exclusion and further to ≤0.016 when *APOE*2 is added, indicating dependence on imputation noise and *APOE*-related homogenization. Together, these patterns support a selection criterion that favors SNPs with RFI ≥ 0.1 in at least one cleaned configuration, stability under Miss_SNP exclusion and *APOE*2 stratification, and reproducible contributions across groups, while excluding markers whose apparent importance is driven by noise or *APOE*-sensitive artifacts. These SNPs not only demonstrate methodological robustness but also point to biologically plausible candidates whose genes align with established AD pathways, warranting functional exploration.

Apart from *APOE*, the genes where the relevant SNPs are located were also explored in terms of function and impact on sAD development (consulted in December 2025^3^). *PRRC2C* (rs2421847) codes for a proline-rich coiled-coil protein (PRRC), which belongs to a family of proteins that are rich in proline amino acids and coiled-coil structures. These are RNA-binding and involved in the formation of stress granules, recently linked to the development of liver cancer (Zhang et al., 2023). Other studies refer to another proline-rich coiled-coil protein (PRRC2A) to be downregulated in astrocytes and microglia in aging mice (Pan et al., 2020). *PPIAP59* (also known as *LOC390956*, rs7245858) encodes a peptidyl prolyl isomerase, this family of proteins has been linked to neurodegenerative diseases and Tau regulation (Liou et al., 2003). Pin1 (another prolyl isomerase) binds to phosphorylated Tau and restores its binding capacities towards microtubules, therefore its expression has been found to be inversely correlated with neurodegeneration in sAD brains (Liou et al., 2003; Lim and Lu, 2005). *PDE7B* (also known as *LOC644135*, rs11154851) encodes an antisense RNA molecule that has been associated with long non-coding RNA family (lncRNA). LncRNAs have been linked to neurodegenerative diseases such as sAD, by regulating gene expression (in particular *BACE1AS*). *BACE1AS* was described as activated in sAD cases, creating an RNA duplex with BACE1 mRNA, allowing the translation of BACE1 mRNA (Ma et al., 2013). *CYCS* (rs1861525) gene encodes for a small heme protein that plays a role in the electron transport chain in mitochondria. The enzyme produced, cytochrome c oxidase (COX) is found in lower levels in sAD brains and platelets, although this is not considered a direct consequence of neurodegeneration (Cardoso et al., 2004). Finally, *AKAP6* gene (rs4296166) encodes an A-kinase anchoring protein. The role of kinases in sAD pathogenesis has been long investigated, involved in Tau hyperphosphorylation, tangle creation, neuroinflammation and cell death (Li et al., 2023; Ansari et al., 2024). While these prioritized SNPs implicate pathways with established relevance to AD pathogenesis (RNA regulation, Tau dynamics, mitochondrial function, kinase signaling), several study constraints temper interpretation and highlight priorities for validation.

The fact that only one *APOE* SNP is present in these ADNI datasets might reduce the overall impact of this analysis. Furthermore, this study is based on moderate sample numbers, due to two reasons: (i). the aim to maintain rigorous sAD standards with biomarker confirmation per updated guidelines in databases where gold-standard neuropathological diagnosis is uniformly guaranteed across subjects (McKhann et al., 2011; Jack et al., 2024) and (ii). the challenges in accessing genomic and corresponding meta data for extraction. More data must be collected and jointly analyzed to further explore and confirm the observations, particularly since sAD subtypes occur at unequal frequencies that may yield imbalanced ratios compromising identification - as with the typical form’s abundance [∼55% by Tau criteria (Ferreira et al., 2020)]. Finally, the lack of information that commonly accompanies the genetic data in sAD genomic databases prevents the creation of machine learning supervised models (e.g., classification), thus requiring continued use of unsupervised approaches like those tested here to uncover genetic subtypes without prior labels. Multimodal models such as HYDRA (Varol et al., 2017), Gene-SGAN (Yang et al., 2024) and even Tijms et al. (2024) that combine genetics with either MRI imaging data or proteomics bring more compelling results to sAD research, fundamentally arguing in favor of these methods to refine sAD subtyping and supporting their prioritization to further characterize this and similar neurodegenerative diseases.

This study serves as an exploratory pilot demonstrating the feasibility of genotypic subtyping in biomarker-confirmed sAD using small curated SNP panels (9–25 SNPs). This approach avoids noise in both subjects and genetic data, while outperforming dilution. While the moderate sample sizes reflect the stringent ADNI filtering prioritizing diagnostic quality over quantity - a strategy aligning with international guidelines (McKhann et al., 2011; Jack et al., 2024) and whose violation has drawn critiques (Escott-Price and Hardy, 2022; Xavier and Pinto, 2025), they enable robust internal replication across panels and algorithms, as evidenced by consistent k = 2 preference and Supplementary Table 3 metrics. The prioritized SNPs (rs2421847, rs7245858, rs11154851, rs4296166) provide a focused starting point for validation and mechanistic studies. While the explored ADNI arrays lack rs429358, rs7412 stratification successfully isolated non-*APOE* signals, demonstrating proxy utility; full *APOE* imputation in expanded cohorts remains ideal. Larger confirmatory cohorts will build on this foundation, particularly for rarer subtypes.

## Conclusion

The accurate diagnosis of sporadic Alzheimer’s disease requires biological confirmation with validated biomarkers. sAD subtypes are well characterized and bring variability into the disease’s manifestation and progression, but genomic interplay in this sporadic form is still poorly integrated in clinical practice. Genomic data available in public databases are commonly under-detailed, leading to several issues in recent GWAS that focus solely on enlarging sampling numbers regardless of diagnosis quality (Escott-Price and Hardy, 2022; Xavier and Pinto, 2025). Moreover, the lack of further characterization of these genomic data precludes the definition of sAD subtypes for other diagnostic criteria, compromising the development of supervised algorithms for classification (for example). Therefore, the application of unsupervised algorithms to genomic data is necessary for sAD subtype exploration.

This study provides novel evidence that small, curated SNP panels (∼9–19 markers) outperform combined sets in sAD clustering, identifying rs2421847 (*PRRC2C*), rs7245858 *(PPIAP59*), rs11154851 (*PDE7B*), and rs4296166 (*AKAP6*) as top contributors resilient to *APOE*2 stratification, noise, and algorithm variation; unveiling binary structure that demands further study and functional validation of these markers. Novel methodological advances include systematic *APOE* exclusion testing and sensitivity analysis, revealing agglomerative clustering’s superior stability over KMeans/DBSCAN in heterogeneous data.

Despite their preliminary nature, these results suggest a new paradigm for sAD research: rather than a unitary disease with varied manifestations, sAD may represent a genetically heterogeneous syndrome comprising distinct subtypes tied to specific molecular pathways. This view aligns with recent multimodal (Varol et al., 2017; Yang et al., 2024) and proteomics (Tijms et al., 2024) studies, opening several research directions: (i). validation in independent biomarker-confirmed cohorts to test SNP generalizability across populations; (ii). multimodal integration with neuroimaging (MRI/PET), CSF biomarkers (p-tau/Aβ), and clinical trajectories to map genetic subtypes onto biological phenotypes; (iii). functional validation via eQTL analysis, pathway enrichment, or cellular/animal models to elucidate subtype mechanisms; (iv). therapeutic stratification, as subtype-specific molecular pathologies may respond better to targeted interventions than current one-size-fits-all trials; and (v). polygenic risk refinement by incorporating subtype-specific SNPs into stratified PRS for improved trajectory prediction and treatment response. If successful, this genotypic foundation promises precision medicine advances in heterogeneous sAD.

## Data Availability

Publicly available datasets were analyzed in this study. This data can be found here: https://adni.loni.usc.edu/.

## Footnotes

1
https://adni.loni.usc.edu/

2
https://www.ensembl.org

3
https://www.ncbi.nlm.nih.gov/gene/
