# Supplementary material for: Mapping Alzheimer’s disease heterogeneity through exploratory unsupervised learning
Source: Front Aging Neurosci. 2026 Jul 16;18:1869804. doi: 10.3389/fnagi.2026.1869804 (PMC13422478; doi:10.3389/fnagi.2026.1869804)

Supplementary Fig. S1. Sankey plots depicting the flow of samples between clusters of different sets of trials. (A) Group 1 to Group 1 + *APOE*2, (B) Group 2 to Group 2 + *APOE*2, (C) Group 2 Miss SNP to Group 2 Miss SNP + *APOE*2 and (D) All SNPs Miss SNP to All SNPs Miss SNP + *APOE*2.

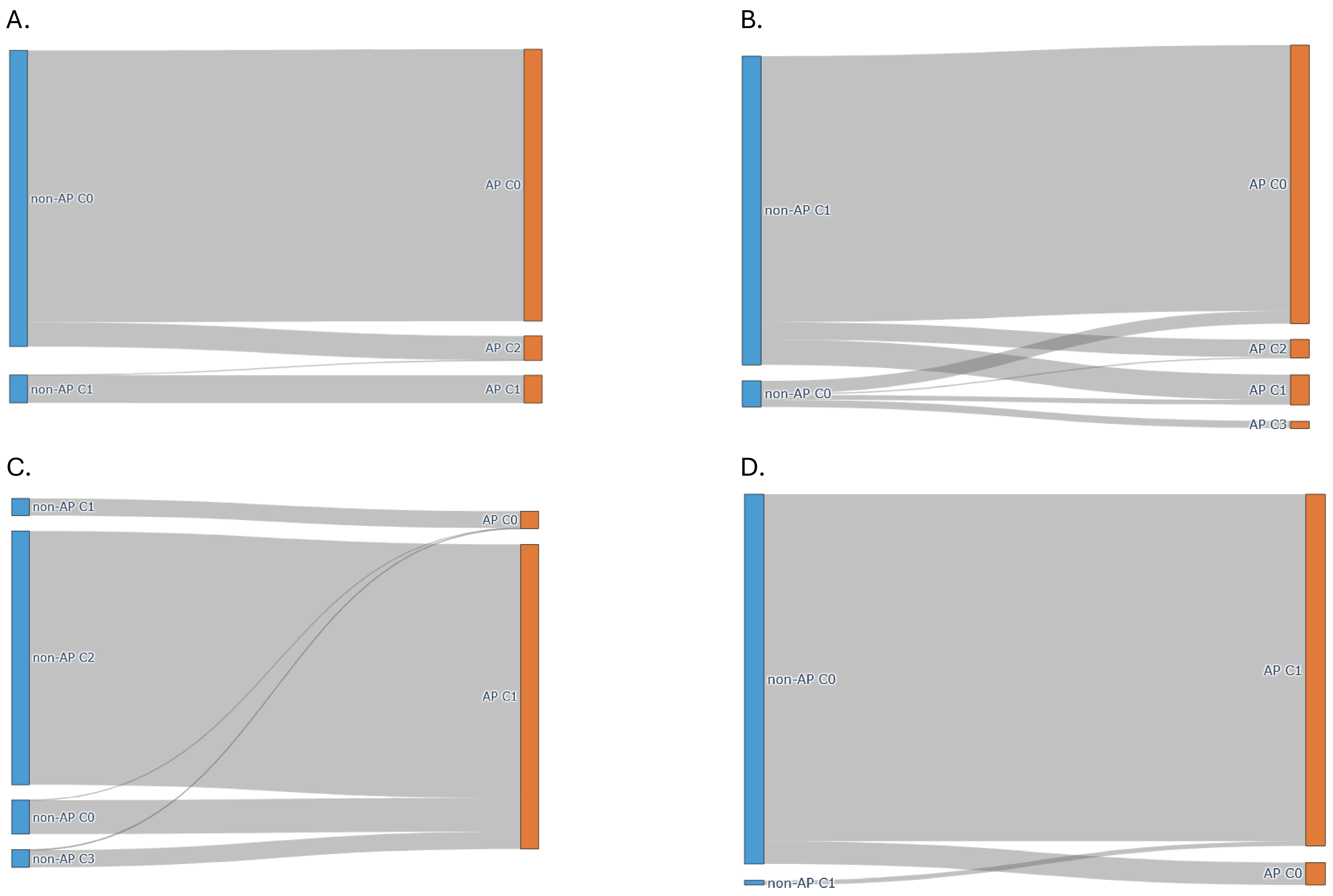

Supplement: Supplementary file 2 [file Data_Sheet_1.pdf]
