# Supplementary material for: Mapping Alzheimer’s disease heterogeneity through exploratory unsupervised learning
Source: Front Aging Neurosci. 2026 Jul 16;18:1869804. doi: 10.3389/fnagi.2026.1869804 (PMC13422478; doi:10.3389/fnagi.2026.1869804)

Supplementary Fig. S2. Co-occurency heatmap of All SNPs Miss SNP (All SNPs) and All SNPs Miss SNP *APOE2* (All SNPs AP) cluster trials. As visible most sample pairs co-occur in the same clusters for both sets (1, darker red).

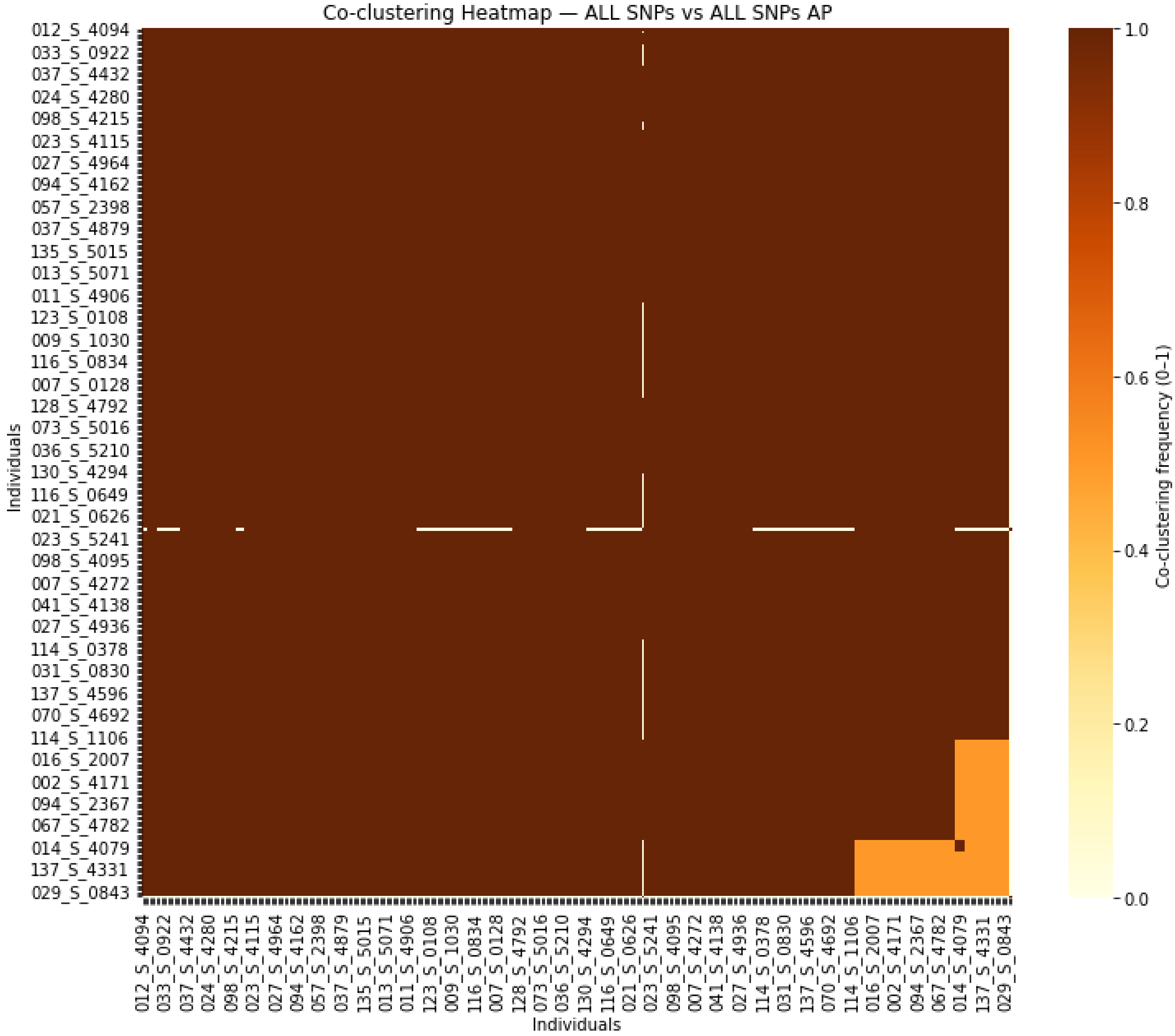

Supplement: Supplementary file 3 [file Data_Sheet_2.pdf]
