# Supplementary material for: Mapping Alzheimer’s disease heterogeneity through exploratory unsupervised learning
Source: Front Aging Neurosci. 2026 Jul 16;18:1869804. doi: 10.3389/fnagi.2026.1869804 (PMC13422478; doi:10.3389/fnagi.2026.1869804)
Supplement: Supplementary file 4 [file Data_Sheet_3.pdf]

```
# -*- coding: utf-8 -*-
"""
Supplementary Material

Mapping Alzheimer’s disease heterogeneity through exploratory unsupervised
learning

This script performs:
1. Merging genotype and phenotype data (ADNI dataset)
2. Selection of Alzheimer's disease cases (diagnosis code = 3)
3. SNP standardization (z-score)
4. Unsupervised clustering:
   - KMeans
   - Agglomerative Clustering
   - Spectral Clustering
   - Gaussian Mixture Models
   - DBSCAN
5. Internal validation of clustering results:
   - Silhouette score
   - Calinski-Harabasz index
   - Davies-Bouldin index
"""

# -----------------------------
# Imports
# -----------------------------

import pandas as pd
import numpy as np

from sklearn.preprocessing import StandardScaler
from sklearn.cluster import KMeans, AgglomerativeClustering,
SpectralClustering, DBSCAN
from sklearn.mixture import GaussianMixture
from sklearn.metrics import (
    silhouette_score,
    calinski_harabasz_score,
    davies_bouldin_score
)

# -----------------------------
# Load phenotype data
# -----------------------------
phenotype = pd.read_csv("ADNI_phenotype_patho.csv")
phenotype.rename(columns={"SampleID": "IID"}, inplace=True)

geno_files = [
    "xxx1_data.csv",
    "xxx2_data.csv",
    "xxx3_data.csv"
]

DIAGNOSIS_CODE = 3


# -----------------------------
# Clustering evaluation
# -----------------------------
def evaluate(X, labels, method, k, iteration):
    """
    Compute internal clustering validation metrics.
    """
    try:
        sil = silhouette_score(X, labels)
```

```python
        ch = calinski_harabasz_score(X, labels)
        db = davies_bouldin_score(X, labels)
    except Exception:
        sil, ch, db = np.nan, np.nan, np.nan

    return {
        "Method": method,
        "k": k,
        "Iteration": iteration,
        "Silhouette": sil,
        "Calinski-Harabasz": ch,
        "Davies-Bouldin": db
    }


# ----------------------------
# Run clustering algorithms
# ----------------------------
def run_clustering(X, k_values=[2, 3, 4, 5], n_iter=3):
    results = []

    for k in k_values:
        for i in range(n_iter):

            # KMeans
            kmeans = KMeans(n_clusters=k, random_state=i, n_init=10)
            labels = kmeans.fit_predict(X)
            results.append(evaluate(X, labels, "KMeans", k, i))

            # Agglomerative
            agg = AgglomerativeClustering(n_clusters=k)
            labels = agg.fit_predict(X)
            results.append(evaluate(X, labels, "Agglomerative", k, i))

            # Spectral Clustering
            spectral = SpectralClustering(
                n_clusters=k,
                affinity="nearest_neighbors",
                random_state=i
            )
            labels = spectral.fit_predict(X)
            results.append(evaluate(X, labels, "Spectral", k, i))

            # Gaussian Mixture
            gmm = GaussianMixture(n_components=k, random_state=i)
            labels = gmm.fit_predict(X)
            results.append(evaluate(X, labels, "GaussianMixture", k, i))

    # DBSCAN (no predefined k)
    dbscan = DBSCAN(eps=2, min_samples=5)
    labels = dbscan.fit_predict(X)

    if len(set(labels)) > 1:
        results.append(evaluate(X, labels, "DBSCAN", None, 0))

    return pd.DataFrame(results)


# ----------------------------
# Main pipeline
# ----------------------------
all_results = []

for file in geno_files:
```

```
    print(f"Processing {file}")

    # Load genotype data
    geno = pd.read_csv(file, sep=";")

    # Merge with phenotype
    data = geno.merge(
        phenotype[["IID", "DIAGNOSIS"]],
        on="IID",
        how="inner"
    )

    # Keep only Alzheimer's disease cases
    data = data[data["DIAGNOSIS"] == DIAGNOSIS_CODE].copy()

    # Extract genotype matrix
    X = data.drop(columns=["IID", "DIAGNOSIS"], errors="ignore")

    # SNP genotypes encoded as 0/1/2 (minor allele dosage)
    # Standardization ensures equal contribution of all SNPs to distance-
based clustering
    X = StandardScaler().fit_transform(X)

    # Run clustering
    results = run_clustering(X)
    results["Dataset"] = file

    # Save per dataset results
    output_file = file.replace(".csv", "_clustering_results.csv")
    results.to_csv(output_file, index=False)

    all_results.append(results)

# Save combined results
pd.concat(all_results).to_csv("all_clustering_results.csv", index=False)

print("Clustering analysis completed.")
```
